# Linking DNRA community structure and activity in a shallow lagoonal estuarine system

**DOI:** 10.3389/fmicb.2014.00460

**Published:** 2014-09-03

**Authors:** Bongkeun Song, Jessica A. Lisa, Craig R. Tobias

**Affiliations:** ^1^Department of Biological Sciences, Virginia Institute of Marine Science, College of William & MaryGloucester Point, VA, USA; ^2^Department of Marine Science, University of ConnecticutGroton, CT, USA

**Keywords:** DNRA, *nrfA*, qPCR, pyrosequencing, diversity

## Abstract

Dissimilatory nitrate reduction to ammonium (DNRA) and denitrification are two nitrate respiration pathways in the microbial nitrogen cycle. Diversity and abundance of denitrifying bacteria have been extensively examined in various ecosystems. However, studies on DNRA bacterial diversity are limited, and the linkage between the structure and activity of DNRA communities has yet to be discovered. We examined the composition, diversity, abundance, and activities of DNRA communities at five sites along a salinity gradient in the New River Estuary, North Carolina, USA, a shallow temporal/lagoonal estuarine system. Sediment slurry incubation experiments with ^15^N-nitrate were conducted to measure potential DNRA rates, while the abundance of DNRA communities was calculated using quantitative PCR of *nrfA* genes encoding cytochrome C nitrite reductase, commonly found in DNRA bacteria. A pyrosequencing method targeting *nrfA* genes was developed using an Ion Torrent sequencer to examine the diversity and composition of DNRA communities within the estuarine sediment community. We found higher levels of *nrfA* gene abundance and DNRA activities in sediments with higher percent organic content. Pyrosequencing analysis of *nrfA* genes revealed spatial variation of DNRA communities along the salinity gradient of the New River Estuary. Percent abundance of dominant populations was found to have significant influence on overall activities of DNRA communities. Abundance of dominant DNRA bacteria and organic carbon availability are important regulators of DNRA activities in the eutrophic New River Estuary.

## Introduction

Sedimentary nitrogen (N) cycling in rivers and estuaries is highly dependent on microbial processes. Nitrate can be dissimilated by denitrification, dissimilatory nitrate reduction to ammonium (DNRA) and anaerobic ammonium oxidation (anammox), depending on prevailing environmental conditions. Denitrification and anammox remove fixed nitrogen by producing dinitrogen (N_2_) gas. In comparison, DNRA retains nitrogen within an ecosystem by recycling nitrate (NO^−^_3_) to ammonium (NH^+^_4_). Denitrification and DNRA both compete for NO^−^_3_ as an electron acceptor and both processes generally rely on organic carbon and sulfide as the source of electrons in anoxic sediments.

DNRA has been found to be an important nitrogen cycling pathway in various aquatic ecosystems including estuaries (Kelly-Gerreyn et al., 2001; An and Gardner, 2002) and salt marshes (Tobias et al., 2001a; Koop-Jakobsen and Giblin, 2010). Geochemical and physical features such as high carbon to NO^−^_3_ ratios, high levels of sulfide, elevated temperature and salinity provide favorable conditions to support DNRA over denitrification in estuarine and coastal sediments (An and Gardner, 2002; Giblin et al., 2010; Dong et al., 2011). However, abundance, composition, and diversity of DNRA communities have not been evaluated as microbial controls of DNRA in an ecosystem.

A diverse group of microorganisms has been shown to use DNRA as an anaerobic respiratory pathway (Simon and Klotz, 2013). DNRA is carried out by fermentative bacteria or by chemolithotrophic bacteria, which oxidize sulfide or other reduced inorganic substrates. The genes and enzymes involved in the DNRA pathway by fermentative bacteria are well characterized (Simon, 2002). However, little is known about chemolithotrophic bacterial DNRA (Giblin et al., 2013).

A pentaheme cytochrome C nitrite reductase (NrfA) is the central enzyme which catalyzes the reduction of nitrite (NO^−^_2_) to NH^+^_4_ (Einsle et al., 1999). The functional gene *nrfA* is present in diverse groups of bacteria including *Proteobacteria, Planctomycetes, Bacteroides*, and *Firmicutes* (Mohan et al., 2004). Some members of the *Epsilonproteobacteria*, such as *Campylobacter* spp. and *Nautilia profundicola*, are capable of DNRA in the absence of *nrfA* genes through the use of a putative reverse hydroxylamine:ubiquione reductase module pathway (Hanson et al., 2013). In addition, respiratory metabolism pathways in DNRA bacteria are diverse, including fermentation, denitrification, anammox, and sulfate reduction (Simon, 2002; Kartal et al., 2007). Due to this metabolic versatility, the diversity and abundance of DNRA bacteria might be greater than other N transforming organisms in sediments. However, DNRA community composition in sediments has only been examined in two previous studies of the Colne River Estuary, based on *nrfA* gene analysis (Takeuchi, 2006; Smith et al., 2007). The limitation of studies examining the diversity and composition of DNRA bacteria is due to the lack of proper molecular methods capable of *nrfA* gene detection in environmental samples.

In order to gain a better understanding of the microbial and geochemical factors affecting DNRA processes, we examined sediment communities along the estuarine gradient of the New River Estuary, North Carolina, USA. The New River Estuary is a shallow and microtidal estuary with high anthropogenic N loading. The mesohaline regions of the estuary were found to have organic and sulfidic sediments, which may support DNRA (Anderson et al., 2013). In addition, Lisa et al. (2014) suggested a linkage between DNRA and anammox in sulfidogenic sediment communities of the estuary. These characteristics make the New River Estuary an ideal place to examine the importance of DNRA in the estuarine N cycle. Sediment slurry incubation experiments with ^15^NO^−^_3_ were conducted to measure potential DNRA rates, while abundance of DNRA communities was quantified using quantitative PCR (qPCR) of *nrfA* genes. A new method utilizing next generation sequencing techniques was developed to examine diversity and composition of *nrfA* genes in the sediment communities of the New River Estuary.

## Materials and methods

### Site description

The New River Estuary is located in southeastern North Carolina, USA. It is a shallow estuary (<5 m deep) with broad lagoons connected by narrow channels. The New River watershed encompasses a 1436 km^2^ area and receives drainage from mostly forested and agricultural lands in the upper regions of the watershed, while the lower estuary is bordered by extensive intertidal wetlands (Burkholder et al., 1997; Mallin et al., 2005). Barrier islands located at the mouth of the estuary prevent tidal exchange, contributing to the 64 day mean flushing time in the estuary (Ensign et al., 2004). Within the watershed are large numbers of industrialized livestock facilities, the City of Jacksonville and the United States Marine Corps Base at Camp Lejeune. The estuary has been classified as “a nutrient sensitive” estuary since 1998, with nitrogen being the limiting nutrient (Mallin et al., 2000). It is a vulnerable ecosystem with various anthropogenic disturbances.

### Sediment sampling

**Figure 1:**
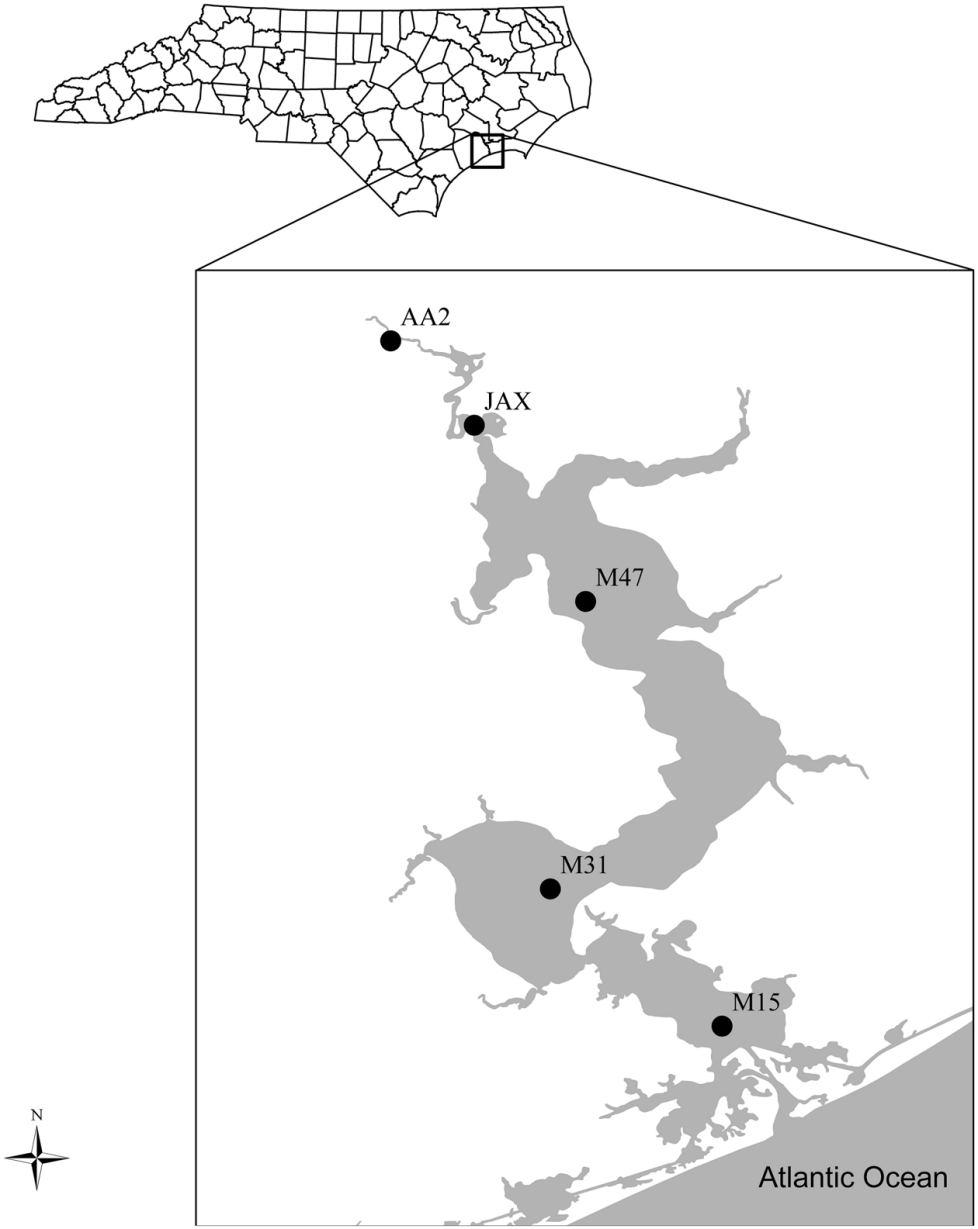
**Sampling Sites in the New River Estuary, NC, USA**. Five sites include an upper estuary site (AA2), two mid-estuary sites (JAX and M47), and two lower estuary sites (M31 and M15).

Sampling along nutrient and salinity gradients from the headwaters to the mouth of the estuary was conducted in April of 2010 (Figure 1). Five sites were examined and included an upstream site AA2 (34°76′N, 77°45′W), two mid-estuary sites JAX (34°73′N, 77°43′W), M47 (34°68′N, 77°39′W), and two lower estuary sites M31 (34°59′N, 77°40′W), M15 (34°55′N, 77°35′W). All samples and measurements were collected in the channel west of the indicated channel markers. Environmental parameters including sediment characteristics and geochemical features of porewater and bottom waters were previously reported by Lisa et al. (2014).

### ^15^N tracer incubations

Sediment slurry incubation experiments with ^15^N tracer, using a modified method of Tobias et al. (2001b), were conducted to measure potential DNRA rates. Sediment slurries containing two grams of homogenized surface sediment (upper 3 cm) were incubated anaerobically in helium-purged Exetainer tubes, following the addition of ^15^NO^−^_3_ tracer (99at%, 200 nmoles). Time series samples were sacrificed by adding saturated ZnCl_2_. DNRA was calculated from the amount of ^15^N tracer measured in the extractable NH^+^_4_ pool. NH^+^_4_ was isolated from the slurry by alkaline acid trap diffusion (Holmes et al., 1998) following the addition of 7 ml of 40 ppt NaCl solution, 0.15 g MgO, and 3 μmoles of unlabeled NH^+^_4_ carrier. The mole fraction excess ^15^NH^+^_4_ (MF) was measured via continuous flow isotope ratio spectrometry using an elemental analyzer interface. The DNRA rate was calculated from:

$$DNRA=\frac{MF{\;}_{15NH4}\;•\;\left[NH4\right]}{MF{\;}_{15NO3}\;•\;t}$$

where *MF* is the ^15^N mole fraction excess of either the extractable ammonium or the added nitrate tracer. The MF_15NH4_ is corrected for the mass of the carrier ammonium. The extractable ammonium concentration [NH_4_] was measured spectrophotometrically using the phenol-hypochlorite method on split sediment slurries, and “*t*” represents the incubation time.

### DNA extraction and quantitative PCR of *nrfA* genes

Sediment DNA was extracted from homogenized sediments using PowerSoil DNA Kit (Mo-Bio Laboratories, Inc., Carlsbad, CA) following the manufacturer's protocol with the following modifications: wet sediment (0.6 g) was used for the extraction and Thermo Savant Fast Prep FP 120 Cell Disrupter (Qbiogene Inc. Carlsbad, CA) was used for cell disruption. qPCR assays of *nrfA* genes were carried out to quantify the abundance of DNRA bacteria in the sediment communities. qPCR reactions were carried out in a volume of 20 uL containing SYBR green using Go-Taq qPCR Master Mix (Promega Corporation, Madison, WI) with 10 ng of DNA and the primers nrfAF2aw (CARTGYCAYGTBGARTA) and nrfAR1 (TWNGGCATRTGRCARTC) (Mohan et al., 2004; Welsh et al., 2014). The primers target heme-binding motifs, which are conserved and diagnostic for the *nrfA* gene. Welsh et al. (2014) tested specificity of the primers with proteobacterial isolates and a soil sample. The expected size of the PCR product is 235–250 bp. PCR conditions were as follows: an initial cycle of 94°C for 10 min; 50 cycles of 94°C for 15 s, 52°C for 45 s, 72°C for 20 s, 80°C for 35 s (data acquisition step); and a dissociation step of 95°C for 15 s, 52°C for 1 min, 95°C for 15 s. Thermal cycling, fluorescent data collection, and data analysis was carried out using ABI Prism 7500 Real Time PCR System Sequence Detection System (Applied Biosystems, Carlsbad, CA).

In order to generate qPCR standards the *nrfA* gene fragment in *Escherichia coli* K-12 was amplified using the primers and PCR conditions described above. The PCR product was cloned using the pGEM-T Easy cloning kit (Promega, Madison, WI) following the manufacturer's instruction. Plasmid DNA was extracted from a culture of recombinant *E. coli* JM109 using the FastPlasmid Mini kit (5 PRIME, Gaithersburg, MD). The plasmid was linearized using the *EcoRI* restriction enzyme and purified using the Wizard SV Gel and PCR Clean-Up System (Promega, Madison, WI). The purified products were quantified using Qubit DNA quantitation assay (Life Technologies, Grand Island, NY) following the manufacturer's instructions. A ten-fold serial dilution of a known copy number of the purified plasmid was prepared for qPCR standards. The qPCR standard has 92.5% identity of DNA sequences with the *nrfA* gene of *Escherichia albertii* in **Figure 3**. qPCR efficiency and the detection limit were evaluated by treating qPCR standards as unknown samples. The qPCR efficiency was 98.9%, although the detection limit was 9 × 10^4^ copies of *nrfA* genes. qPCR inhibition was not apparent based on an additional test using a mixture of qPCR standards and sediment DNA.

### Ion torrent PGM sequencing of *nrfA* genes

Composition and diversity of *nrfA* genes in sediment samples were examined with a barcode pyrosequencing method using the Ion Torrent PGM sequencer. PCR was conducted in duplicate with each 20 μL sample reaction containing the nrfA2Faw and nrfAR1 primers modified to include 8-bp barcode (forward primers) (Hamady et al., 2008) and adapter sequences for the Ion Torrent PGM sequencer (Life Technologies, Grand Island, NY). The primer sequences used for pyrosequencing are listed in Supplementary Table 1. PCR reactions were carried out using Platinum PCR Supermix (Life Technologies, Grand Island, NY) with the following PCR cycle: an initial 5 min at 94°C, 40 cycles of 94°C for 30 s, 52°C for 45 s, and 72°C for 20 s, followed by 5 min at 72°C. PCR results, generating 290 base pair fragments, were checked by running an aliquot on a 2% agarose gel. Duplicate reactions were combined, and amplicons were purified using UltraClean GelSpin DNA Purification Kit (Mo-Bio, Carlsbad, CA). The concentration of purified PCR products was measured using a 2200 TapeStation instrument and D1K reagents (Agilent Technologies, Santa Clara, CA) following the manufacturer's instruction. Pyrosequencing was conducted using an Ion Torrent PGM sequencer with barcode samples pooled on 316 chips, following the Ion Torrent 400 bp sequencing kit protocol (Life Technologies, Grand Island, NY).

### Bioinformatic analysis of *nrfA* sequences

The bioinformatic pipeline of *nrfA* gene pyrosequences is outlined in Supplementary Figure 1. The pipeline contains easy-to-use web based programs and computer based programs. The FastQ file was downloaded from the Torrent Server after primary base calling was conducted using Torrent Suite v3.0 software (Life Technologies). The RDP Pipeline Initial Process (https://pyro.cme.msu.edu/init/form.spr) was used to sort the *nrfA* sequences in 5 libraries based on the barcode sequences. Primer sequences were trimmed, and sequences shorter than 200 bp and lower than 25 quality score were removed. Acacia (Bragg et al., 2012) was used to de-noise the trimmed sequences, and a chimera check was performed using UCHIME in the FunGene Pipeline (http://fungene.cme.msu.edu/FunGenePipeline/chimera_check/form.spr) (Fish et al., 2013). The selected sequences were translated and compared to NrfA reference sequences using the FunGene Pipeline Frambot tool (http://fungene.cme.msu/edu/FunGenePipeline). A total of 383 sequences was used as reference NrfA sequences after trimming and dereplicating 1690 sequences available in the FunGene repository (http://fungene.cme.msu.edu). The Frambot translated sequences were visually inspected to detect and to remove frame-shift errors. PRINSEQ (http://edwards.sdsu.edu/cgi-bin/prinseq/prinseq.cgi) (Schmieder and Edwards, 2011) was used to rename each sequence ID corresponding to the sampling sites. Sequences with renamed IDs (valid sequences) were used to examine diversity of NrfA sequences and compare the composition of DNRA communities.

In order to reduce sequence redundancy in diversity computation, identical NrfA sequences were dereplicated using PRINSEQ (Schmieder and Edwards, 2011). Unique NrfA sequences in each sediment community were aligned by MUSCLE (Edgar, 2004) in MEGA 5.2 (Tamura et al., 2011). The Protdist program in Phylogeny Inference Package (PHYLIP) (Felsenstein, 1989) was used to generate a distance matrix of aligned NrfA sequences with Kimura's method. Rarefaction, richness estimates, and diversity indices were computed based on a distance matrix using DOTUR (Schloss and Handelsman, 2005). Pielou's evenness was calculated by dividing the Shannon diversity index (H) by the natural log (Ln) of total number of operational taxonomic units (OTUs) (Pielou, 1966). In order to select protein distance for OTU determination, a total of 123 NrfA reference sequences from 123 species and 82 genera were analyzed using DOTUR. A protein distance of 0.1 was found to be the sub-genus level cutoff, while a distance of 0.2 was at the genus level. The OTUs were determined based on 0.1 protein distance cutoff, which is approximately 90% amino acid sequence identity. OTUs, determined with 90% amino acid sequence identity, were used in other pyrosequencing analyses of functional genes involved in nitrogen cycling pathways (Mao et al., 2013, 2011; Pereira E Silva et al., 2013).

The composition of sedimentary DNRA communities at the five sites was compared using CD-HIT (Li and Godzik, 2006) by clustering valid NrfA sequences that shared more than 90% identities. For phylogenetic analysis, a representative OTU sequence from each cluster was aligned through MEGA 5.2 (Tamura et al., 2011) using MUSCLE (Edgar, 2004). Normalized weighted UniFrac (Lozupone et al., 2006) was conducted for PCoA analysis to compare differences among the five DNRA communities. The NrfA sequences, found in only one community, were used to compute percent abundance of endemic sequences and OTUs in each community. In addition, the OTUs containing more than 1% of total NrfA sequences in each community were defined as dominant OTUs (or sequences), and percent abundance of the dominant sequences were calculated. The representative sequences of dominant OTUs, along with the reference NrfA sequences, were also used for phylogenetic analysis. Bootstrap analysis of 1000 repetitions was used to estimate reliability of phylogenetic reconstruction with 50% support threshold. In addition, a heat map was constructed with the percent abundance of dominant OTUs using Microsoft Excel.

### Statistical analysis

Pearson's correlation and analysis of variance (ANOVA) were conducted to identify relationships among DNRA rates, *nrfA* gene abundance, diversity, composition, and environmental parameters using the StatPlus program (AnalystSoft Inc.). Canonical correspondence analysis (CCA) was also performed to examine covariance among environmental variables and the dominant OTUs.

### Nucleotide sequence accession numbers

The pyrosequences of *nrfA* genes were deposited in the ENA Short Read Archive under submission number PRJEB6248.

## Results

### Physical and geochemical characteristics of the new river estuary

Geochemical and physical characteristics of bottom water and sediment samples at the five sites are reported in Table 1. The data were obtained from the seasonal study of anammox communities (Lisa et al., 2014). Bottom water salinity ranged from 9.1 to 33.6 ppt along the estuary. AA2 was designated as an oligiohaline site, JAX and M47 were both mesohaline, and M31 and M15 were situated in the polyhaline reaches of the estuary. Bottom water NH^+^_4_ concentration was higher than NO^−^_3_ concentration at all the sampling sites. Higher concentrations of H_2_S in porewater were measured in the mesohaline sites JAX and M47 compared to other sites. Sediment % organic content and extractable NH^+^_4_ were also higher in these mesohaline sediments (Table 1).

**Table 1:** **Physical and geochemical characteristics measured at 5 sampling sites in the New River Estuary**.

**Sampling site**	**Bottom water**			**Sediment**			
	**Salinity ppt**	**NO^−^_3_ μM**	**NH^+^_4_ μM**	**% organic**	**H_2_S μM**	**NO^−^_x_ μM**	**Extractable NH^+^_4_ μmol g^−1^**
AA2	9.1	0.44	7.8	15.57	0.90	0.26	0.06
JAX	17.8	0.25	0.75	17.98	486.90	0.23	0.21
M47	16.4	0.37	1.18	18.95	249.87	0.54	0.28
M31	27.0	0.47	1.45	10.19	3.21	0.33	0.20
M15	33.6	0.62	1.32	0.33	0.20	0.72	0.04

### Abundance and activities of DNRA communities

Potential rates of DNRA at the five sites ranged from 2.15 to 25.09 nmoles N g^−1^ h^−1^ (Table 2). Higher DNRA rates (>20 nmoles N g^−1^h^−1^) were measured in the sediments from JAX, M47, and M31, while the lowest rate was found at M15. Abundance of DNRA communities based on *nrfA* gene detection agreed with rate measurements as higher abundance of *nrfA* was also measured in the sediments of JAX, M47, and M31. Among geochemical characteristics measured at each site, % organic content and extractable NH^+^_4_ in sediments positively correlated with *nrfA* gene abundance and DNRA rates with no significant statistical support (Supplementary Table 2).

**Table 2:** **Potential rates of DNRA and *nrfA* gene abundance in the five sediment communities of the New River Estuary**.

**Sampling site**	**DNRA rates nmoles N g^−1^ h^−1^**	***nrfA* gene copies g^−1^**
AA2	13.8 ± 1.8	7.72 × 10^8^ ± 4.76 × 10^7^
JAX	20.7 ± 0.02	2.58 × 10^9^ ± 6.27 × 10^7^
M47	22.6 ± 1.0	1.56 × 10^9^ ± 2.68 × 10^8^
M31	25.1 ± 3.4	2.18 × 10^9^ ± 3.16 × 10^8^
M15	2.2 ± 0.8	2.31 × 10^8^ ± 2.50 × 10^7^

### Diversity of DNRA communities based on *nrfA* gene pyrosequencing

A total of 50,882 sequences was obtained following the initial process of filtering the sequences. Total numbers of sequences per site are listed in Table 3. Denoising and chimera check yielded 47,591 sequences, which were translated to amino acid sequences using Frambot. After manually removing frame shift errors, 46,165 valid NrfA sequences were used to compare composition and diversity of DNRA communities (Table 3). After dereplicating identical NrfA sequences, approximately 2000 sequences were classified as unique to each site.

**Table 3:** **Number of *nrfA* sequences filtered during different steps of bioinformatic analysis**.

**Sampling site**	**Number of sequences**					
	**Initial RDP process**	**Denoise/Chimea Check**	**Frambot^a^**	**Valid^b^**	**Dominant^c^ (%)**	**Endemic^d^ (%)**
AA2	7,918	7,557	7,483	7,346	42.3	30.9
JAX	8,821	7,940	7,910	7,852	50.7	11.7
M47	13,870	13,464	13,130	12,952	46.8	13.6
M31	11,197	10,243	10,218	10,000	50.2	16.7
M15	9,076	8,387	8,330	8,015	38.6	39.8

a Frambot converted DNA sequences into amino acid sequences and identified nrfA genes based on the reference sequences.

b Valid sequences were defined as amino acid sequences without frame-shift errors.

c Dominant sequences were defined as an OTU comprising of more than 1% of total NrfA sequences in each community.

d *Endemic sequences were detected at only one site*.

Richness and diversity of each DNRA community were evaluated based on the unique NrfA sequences (Table 4). Chao1 and ACE estimates showed highest richness of NrfA in AA2 sediment, while the lowest was in M47 community. Shannon index showed the highest diversity of NrfA in AA2 and the lowest in M47. The highest sequence evenness was found at M15, where the lowest DNRA rate was measured (Table 2). The M31 community, with the highest DNRA rate, was found to have the lowest evenness. Among the environmental variables, extractable NH^+^_4_ had a significant and negative correlation with NrfA sequence diversity (*r* = −0.94 and *p* = 0.016, Supplementary Table 2). There was no clear trend between salinity gradients and NrfA diversity, although highest richness and diversity of NrfA sequences were observed at AA2, the oligohaline site (Table 4).

**Table 4:** **Estimates of sedimentary NrfA sequence richness and diversity in the New River Estuary**.

**Sampling site**	**Unique**	**OTUs^a^**	**Chao1^a^**	**ACE^a^**	**Shannon^a^**	**Evenness^b^**
AA2	2,052	1,012	1,958.6	2,162.3	6.507	0.940
JAX	1,714	827	1,569.5	1,638.0	6.298	0.938
M47	2,022	799	1,237.8	1,381.5	6.188	0.926
M31	2,210	1,005	1,897.8	2,106.9	6.360	0.920
M15	2,157	933	1,489.9	1,603.5	6.446	0.943

a Richness and diversity were determined based on 0.1 protein distance.

b *Evenness was calculated by dividing Shannon index by Ln (OTUs)*.

### Comparison of DNRA community composition based on *nrfA* gene pyrosequencing

**Figure 2:**
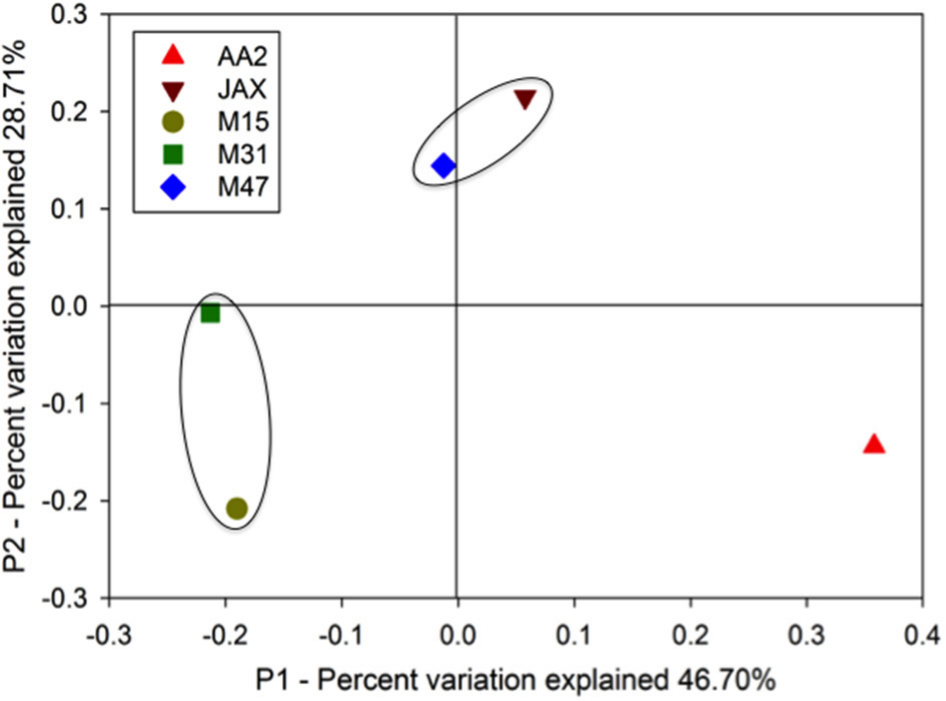
**Weighted and normalized PcoA plot of the DNRA communities in the New River Estuary**. Unifrac analysis was conducted with representative NrfA OTUs in the five sediment communities. The black circles indicate the communities sharing higher composition similarities.

Based on 90% identity as a cutoff of sub-genus level, the valid sequences were clustered into OTUs, and a representative sequence from each OTU was used for normalized weighted Unifrac analysis. Figure 2 shows the spatial variation of the DNRA communities with 75.41% percent variation explained (sum of the first and second PCoA principal coordinates). The AA2 community was the most distinct of the DNRA communities. The composition of JAX and M47 communities were more similar to each other than to other communities, while M31 and M15 communities also clustered together. Salinity was a significant environmental factor (*p* < 0.05) segregating these communities along the P1 coordinate, while bottom water NO^−^_3_ concentrations, porewater H_2_S and extractable NH^+^_4_ became significant variables (*P* < 0.05) along the variation of the P2 coordinate.

Cluster analysis revealed that endemic sequences comprised less than 17% of total NrfA sequences in JAX, M47, and M31 communities and more than 30% in AA2 and M15 (Table 3). Relative percent of endemic sequences (sequences found in only one community) was higher in AA2 and M15 communities, where rates were lowest, than in the mid estuarine communities. The highest relative percent of endemic sequences was found in the M15 community with 39.8%, while the JAX community had the lowest with 11.7% (Table 3). The numbers of endemic OTUs in each community significantly and positively correlated with diversity of NrfA sequences (*r* = 0.95, *p* < 0.05). Most of the OTUs with endemic sequences were rare OTUs (≤0.1% of total sequence) or low abundant OTUs (between 0.1 and 1% of total sequences) in each community. Percent abundance of endemic sequences was significantly and negatively correlated with the DNRA rates and *nrfA* gene abundance (*r* = −0.93 and *r* = −0.93, respectively). In contrast, the dominant OTUs were more abundant in the mid estuarine communities than the AA2 and M15 communities. More than 50% of total NrfA sequences in JAX and M31 sites were clustered with the dominant OTUs (Table 3). Percent abundance of dominant sequences had significant and positive correlations with activities and abundance of DNRA communities (*r* = 0.92 and *r* = 0.99, respectively).

### Comparison of dominant NrfA OTUs in sediment communities

**Figure 3:**
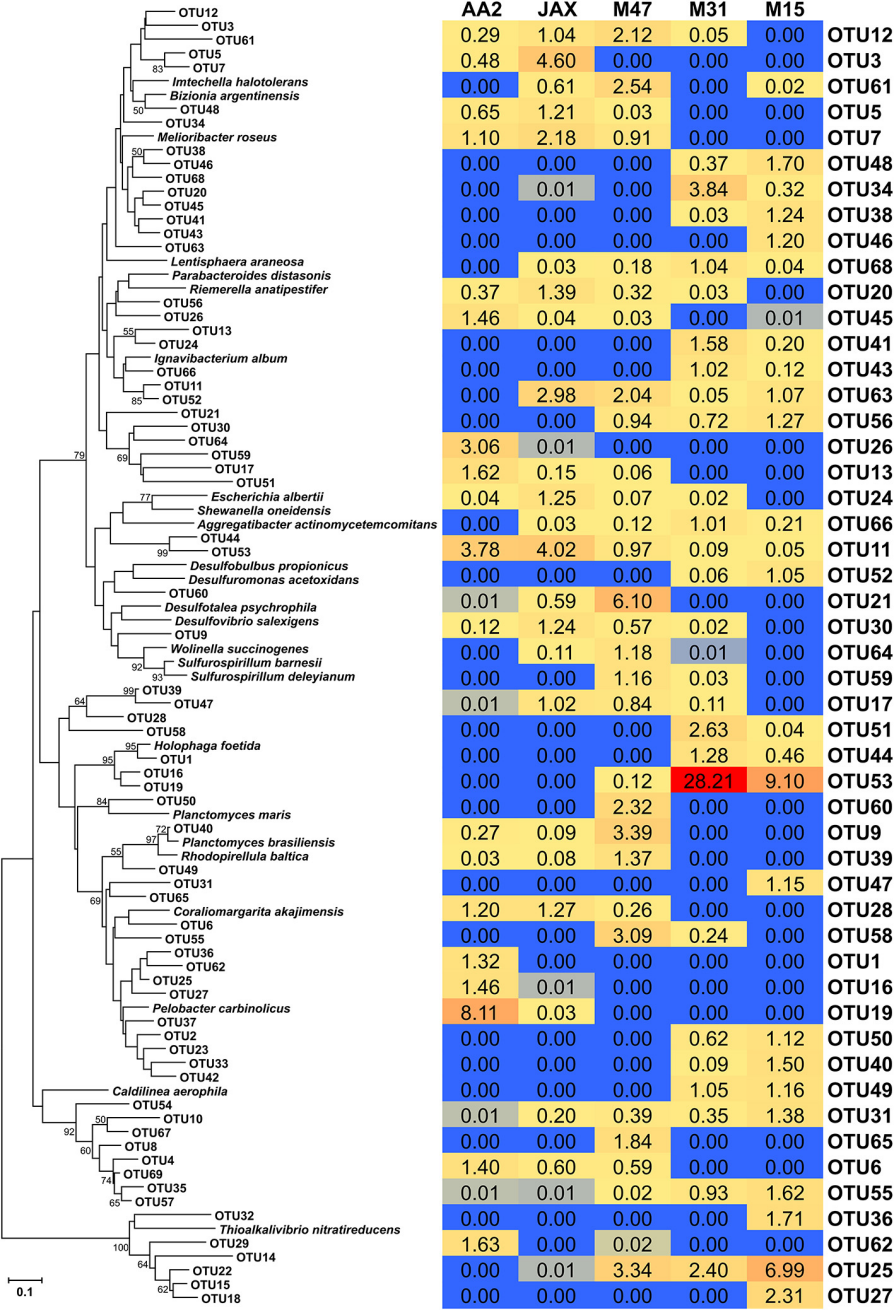
**Phylogenetic tree and heat map of dominant NrfA OTUs in the New River Estuary**. Dominant OTUs were defined as the OTUs containing more than 1% of total number of NrfA sequences in each community. Neighbor-joining tree was constructed from amino acid sequences and bootstrap analysis with 1000 replicates used to estimate confidence. Bootstrap values of >50% were listed in the tree. The heat map was constructed in Microsoft Excel based on the percent relative of each OTU in each sediment community.

A total of 69 dominant OTUs was found in five sediment communities. The sequences associated with the dominant OTUs in each community were included in the heat map analysis, although this accounted for less than 1% of sequence abundance (Figure 3). A representative sequence of each OTU was phylogenetically compared with NrfA sequences found in bacterial isolates (Figure 3). The AA2, JAX, and M15 communities had higher numbers of dominant OTUs (18, 20, and 19, respectively) than the M47 and M31 communities, which had 16 and 13 dominant OTUs, respectively (Supplementary Table 3). Most of dominant OTUs were found in more than one community, with the exception of 10 OTUs. OTU1 and OTU2 were endemic at AA2, while OTU27, OTU36, OTU37, OTU46, and OTU47 were only found in the M15 community. OTU60 and OTU65 were endemic at M47, and OTU54 was only present at M31. In contrast, OTU11, OTU23, OTU31, and OTU 55 were cosmopolitan OTUs commonly present in all five sediment communities, although none of them were predominant in any of the five sediment communities. OTU11 and OTU23 were dominant in the AA2 and JAX communities, but not in other communities. OTU31 and OTU55 were only dominant in M15.

Among 69 OTUs, OTU53 was the most abundant member of M31 (28.2%) and M15 (9.1%), but accounted for less than 1% at M47 and was not detectable at AA2 and JAX (Figure 3). Phylogenetic analysis showed that OTU53 was closely related to NrfA found in *Escherichia albertii, Shewanella oneidensis*, and *Aggregatibacter actinomycetemcomitans* (Figure 3). The most abundant member in M47 community was OTU21 (6.1%), while OTU35 accounted for 5.6 and 14.6% at JAX and M47, respectively. NrfA sequences found in bacterial isolates did not cluster with these OTUs (Figure 3). OTU19, which is closely related to *Holophaga foetida* NrfA, was most abundant, composing 8.1% in the AA2 community, but contributed less than 1% in JAX, and not detected in other sites (Figure 3). Phylogenetic analysis showed that OTU9, dominant in M47, was closely associated with *Sulfurospirillum deleyianum* and OTU32, the dominant member of JAX and M47 communities, was related to *Thioalkalivibrio nitratireducens* (Figure 3).

**Figure 4:**
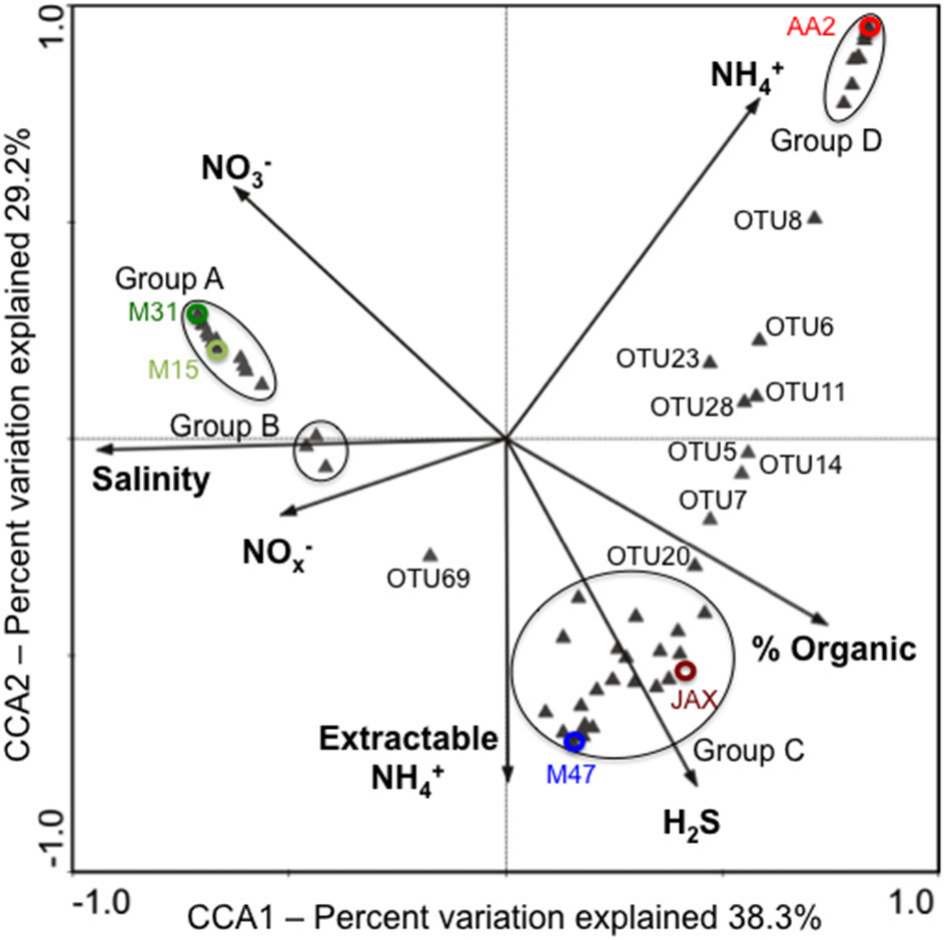
**CCA ordination plot deciphering the relationship between dominant NrfA OTUs and environmental variables in the New River Estuary**. Color circles represent the ordinates of each community based on dominant OTUs. Some of dominant OTUs were clustered in groups as indicated with black circles. Group A contains OTUs 27, 31, 34, 36, 37, 38, 40, 41, 42, 43, 44, 46, 47, 48, 49, 50, 51, 52, 53, 54, 55, 56, 57, 66, and 68. Group B has OTUs25, 31, and 56. Group C carries OTUs 3, 4, 9, 12, 17, 21, 24, 29, 30, 32, 33, 35, 39, 58, 59, 60, 61, 63, 64, 65, and 67. Group D includes OTUs 1, 2, 10, 13, 15, 16, 18, 19, 22, 26, 45, and 62.

CCA with dominant OTUs showed that the first two CCA axes (CCA1 and CCA2) explained 67.5% of the cumulative variance of the DNRA communities in the New River Estuary (Figure 4). Different environmental variables affected relative abundance of dominant OTUs in sediment communities. Group A was composed of 24 OTUs that were dominant members of M31 or M15 communities. Salinity significantly and positively influenced the abundance of OTU49, OTU50, and OTU55 within Group A. OTU49 and OTU50 were closely related to *Planctomyces* spp. (Figure 3). Group B was made up of three OTUs (25, 31, and 56) found in three to five communities. Porewater NO^−^_x_ concentrations had a positive significant correlation with this group. The OTUs in Group C were dominant members of JAX or M47 communities. Sulfide concentration was shown to have significant and positive influences on 10 OTUs (17, 20, 24, 29, 30, 32, 35, 63, and 67) within this group. None was closely related to NrfA sequences found in bacterial isolates. Group D was composed of 12 OTUs (1, 2, 10, 13, 15, 16, 18, 19, 22, 26, 45, and 62) found primarily in the AA2 community (Figure 3). They had a significant and positive correlation with bottom water NH^+^_4_ concentration. Among these dominant OTUs, OTU1, OTU16, and OTU19 were closely associated with *H. foetida*, while OTU2 showed high similarity to *Pelobacter carbinolicus*. Together, the CCA and phylogenetic analyses revealed that different environmental variables contributed to the presence of the different dominant members in DNRA communities at oligohaline, mesohaline, and polyhaline reaches of the New River Estuary.

## Discussion

Two dissimilatory NO^−^_3_ reduction pathways, DNRA and denitrification, influence the recycling and removal of fixed N in an aquatic ecosystem. We found that sedimentary DNRA activities were higher than the reported denitrification activities collected from AA2, M47, M31, and M15 during the same time (Supplementary Figure 2; Lisa et al., 2014). DNRA was responsible for 44–74% of sedimentary dissimilatory NO^−^_3_ reduction. This demonstrates that DNRA was the major dissimilatory NO^−^_3_ reduction process in the New River Estuary, as shown in other estuarine ecosystems (Kelly-Gerreyn et al., 2001; Tobias et al., 2001a,b; An and Gardner, 2002). DNRA rates measured in the New River Estuary are comparable to those reported in shallow coastal systems and salt marshes (Sørensen, 1978; Gardner and McCarthy, 2009; Koop-Jakobsen and Giblin, 2010).

Positive correlation between DNRA rates and extractable NH^+^_4_ in sediments indicates that DNRA is an important process for sediment NH^+^_4_ flux, as well as mineralization. Higher DNRA rates were measured in the mid estuarine sites where H_2_S concentration and % organic contents in sediments were elevated. High H_2_S may inhibit nitrification and denitrification, which results in a greater contribution of DNRA to dissimilatory NO^−^_3_ reduction processes. This was also shown in a shallow estuary in southern Texas (An and Gardner, 2002). In addition, H_2_S can be utilized as an electron donor by DNRA bacteria. Two dominant OTUs in JAX and M47 were closely related with *S*. *deleyianum* and *T*. *nitratireducens*. *S. deleyianum* is a mixotrophic bacterium that can utilize organic carbon and H_2_S as electron sources (Eisenmann et al., 1995), while the obligate chemolithoautotrophic bacterium, *T. nitratireducens*, reduces NO^−^_3_ coupled to sulfur oxidation (Tikhonova et al., 2006). The presence of *Sulfurosprillum* spp. and *Thioalkalivibrio* spp. suggests the use of sulfur compounds as reducing power for dissimilatory NO^−^_3_ reduction in these sediment communities.

The abundance of bacteria capable of DNRA can be an important microbial regulator for the respiratory process in aquatic ecosystems. However, quantification of DNRA bacteria based on *nrfA* gene detection has had limited success. To our knowledge Dong et al. (2009) is the only other study that measured three subsets of *nrfA* gene abundance in the Colne River Estuary. The new primer combination used in this study was able to amplify *nrfA* genes in all five of the sediment samples, even though more than 9x10^4^ copies of *nrfA* genes were required for proper detection. This high detection limit might be due to degeneracy of PCR primers. qPCR success allowed quantification of total abundance of *nrfA* carrying bacteria in estuarine sediments communities, which were then compared with DNRA activities. This is the first study reporting a co-occurrence of higher DNRA rates in the sediment communities with higher *nrfA* gene abundance, and suggests the abundance of DNRA bacteria can act as an important microbial control in estuarine sediments. In addition, *nrfA* gene abundance may have the potential to be used as a genetic proxy of DNRA potential in sediments.

Composition and diversity of DNRA bacteria can be another microbial regulator of DNRA in aquatic sediments. Differences in DNRA community composition were found along the salinity gradient of the New River Estuary. Takeuchi (2006) reported a similar trend in the Colne River Estuary. Endemic populations accounted for 10–40% of the sediment communities in the New River Estuary. The increased numbers of an endemic population supported higher diversity of NrfA sequences, but negatively influenced the DNRA activities. This finding leads us to hypothesize that selected members of DNRA communities become predominant and responsible for overall community activities. We found that relative abundance of dominant populations had a significant positive correlation with the rates and abundance of DNRA communities in the New River Estuary. Bulow et al. (2008) and Francis et al. (2013) demonstrated the presence of a few dominant OTUs in denitrifying communities along the salinity gradient of the Chesapeake Bay, based on the *nirS* gene analysis. The dominant *nirS* OTUs were also highly expressed in the mesohaline and polyhaline sediment communities (Bulow et al., 2008), which supports the importance of dominant populations in *in situ* community activities. These findings readdress the importance of dominant population and their roles in overall community activities.

Dominant DNRA populations were influenced by different geochemical and physical parameters in the New River Estuary. The dominant members in JAX and M47 communities, positively influenced by H_2_S, might be able to oxidize both organic carbon and sulfide as observed in *S. deleyianum* (Eisenmann et al., 1995). Alternatively, DNRA communities in JAX and M47 were more tolerable and adaptive to high sulfidogenic conditions. The dominant members in the oligohaline AA2 community were positively correlated with bottom water NH^+^_4_ concentration, while the members in M31 and M15 were influenced by salinity. This suggests that the dominant populations of each community are well adapted to different environmental conditions present in their unique environments. Sun et al. (2013) reported 13 dominant OTUs were mainly responsible for changes in metal and hydrocarbon contaminants in estuarine sediments based on 16S rRNA gene pyrosequencing analysis. Abundance of different dominant populations in each community strongly influences overall community activities, while geochemical and physical conditions affect the composition of DNRA communities in the New River Estuary. This study reveals that the relative abundances of dominant populations serves as an important microbial control for community activities, while the abundance of endemic populations is an important factor for DNRA bacterial diversity in estuarine sediments.

### Conflict of interest statement

The authors declare that the research was conducted in the absence of any commercial or financial relationships that could be construed as a potential conflict of interest.
