# Supplementary material for: Linking DNRA community structure and activity in a shallow lagoonal estuarine system
Source: Front Microbiol. 2014 Sep 3;5:460. doi: 10.3389/fmicb.2014.00460 (PMC4153293; doi:10.3389/fmicb.2014.00460)
Supplement: Supplementary file 1 [file Presentation1.PDF]

Supplementary Figure 1. Bioinformatic pipeline of *nrfA* pyrosequence analysis.

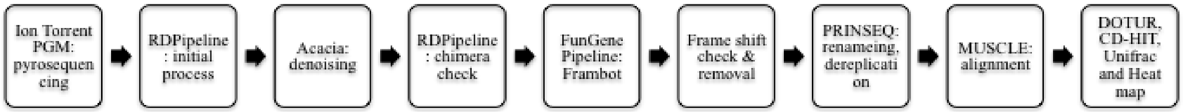

Supplementary Figure 1

Supplementary Figure 2. Comparison of DNRA and denitrification rates measured in five sediment communities in the New River Estuary. The denitrification rates were obtained from Lisa et al (2014).

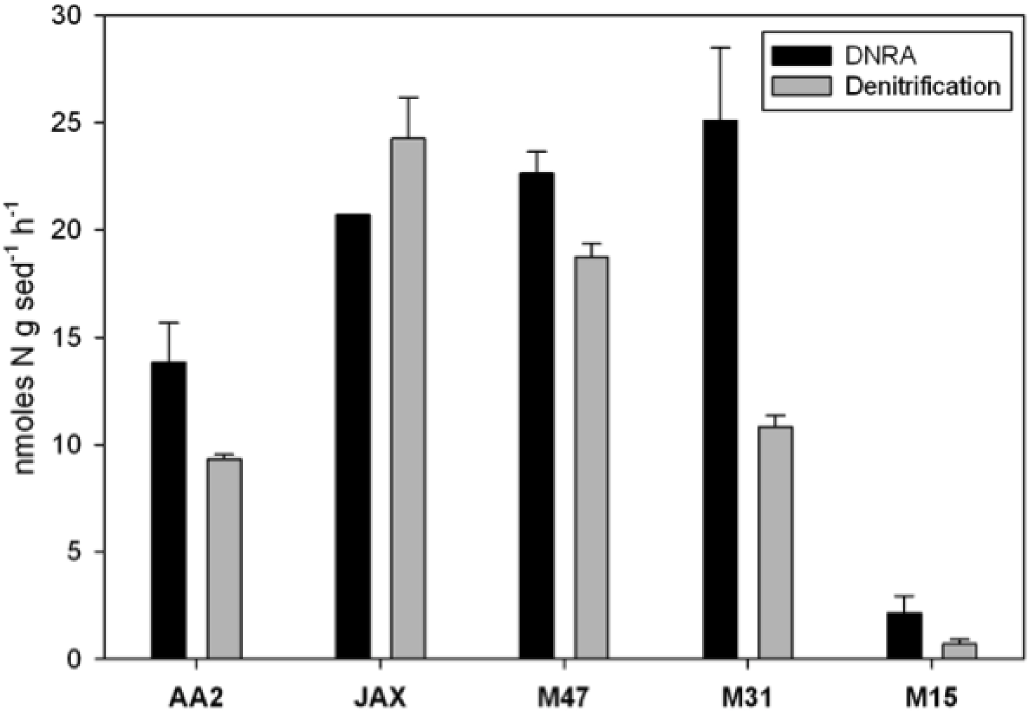

Supplementary Figure 2
