# Supplementary material for: Linking DNRA community structure and activity in a shallow lagoonal estuarine system
Source: Front Microbiol. 2014 Sep 3;5:460. doi: 10.3389/fmicb.2014.00460 (PMC4153293; doi:10.3389/fmicb.2014.00460)
Supplement: Supplementary file 2 [file DataSheet1.PDF]

Supplementary Table 1. Primers used for *nrfA* gene pyrosequencing

Primer name	Direction	
nrfA2F-A-M351	Forward	**CCATCTCATCCCTGCGTGTCTCCGACTCAG** *ATCGGCTA* CARTGYCAYGTBGARTA
nrfA2F-A-M352	Forward	**CCATCTCATCCCTGCGTGTCTCCGACTCAG** *ATCGTACG* CARTGYCAYGTBGARTA
nrfA2F-A-M353	Forward	**CCATCTCATCCCTGCGTGTCTCCGACTCAG** *ATCGTAGC* CARTGYCAYGTBGARTA
nrfA2F-A-M354	Forward	**CCATCTCATCCCTGCGTGTCTCCGACTCAG** *ATCGTTCC* CARTGYCAYGTBGARTA
nrfA2F-A-M355	Forward	**CCATCTCATCCCTGCGTGTCTCCGACTCAG** *ATCGTTGG* CARTGYCAYGTBGARTA
nrfAR1-triP1	Reverse	**CCTCTCTATGGGCAGTCGGTGAT** TWNGGCATRTGRCARTC

Sequences in bold fonts are the PGM adapter.
Sequences in italic fonts are the 8-bp barcode.
Sequences in regular fonts are the *nrfA* primers.

Supplementary Table 2. Pearson's correlation analysis of DNRA rates, abundance, diversity and environmental variables

	Salinity	NO_3^-	NH_4^+	%organic	H_2S	NO_x^-	Extractable NH_4^+
DNRA rate	-0.392	-0.704	-0.197	0.739	0.412	-0.605	0.855
nrfA gene abundance	-0.190	-0.796	-0.437	0.606	0.674	-0.639	0.786
Richness[1]	-0.181	0.134	0.633	-0.084	-0.488	-0.629	-0.451
Diversity[2]	0.067	0.578	0.682	-0.534	-0.670	-0.075	-0.944*
Evenness	-0.070	0.187	0.369	-0.285	0.019	0.139	-0.743
Dorminant sequences	-0.212	-0.766	-0.400	0.625	0.597	-0.638	0.817
Endemic sequences	0.339	0.845	0.418	-0.768	-0.705	0.515	-0.940*

*indicates $P<0.05$.

[1]Chao1 estimate was used to represent the richness of NrfA sequences in each community.

[2]Shannon index represented diversity of NrfA sequences in each community.

Supplementary Table 3. Number of dominant and endemic NrfA OTUs in the five sediment communities of the New River Estuary

Sampling site	Dominant OTUs	Endemic OTUs
AA2	18	678
JAX	20	339
M47	16	310
M31	13	548
M15	19	590
